# Proposal of discontinuation criteria of atezolizumab plus bevacizumab after curative conversion therapy for unresectable early-to-intermediate-stage hepatocellular carcinoma: a multicenter proof-of-concept study

**DOI:** 10.1007/s00535-025-02233-z

**Published:** 2025-03-07

**Authors:** Tomoko Aoki, Masatoshi Kudo, Naoshi Nishida, Kazuomi Ueshima, Kaoru Tsuchiya, Toshifumi Tada, Masahiro Morita, Hirokazu Chishina, Masahiro Takita, Satoru Hagiwara, Hiroshi Ida, Yasunori Minami, Hidekatsu Kuroda, Noriaki Nakamura, Atsushi Hiraoka, Tetsu Tomonari, Joji Tani, Atsushi Naganuma, Satoru Kakizaki, Chikara Ogawa, Takeshi Hatanaka, Toru Ishikawa, Kazuhito Kawata, Atsushi Takebe, Ippei Matsumoto, Masaaki Hidaka, Masayuki Kurosaki, Takashi Kumada, Namiki Izumi

**Affiliations:** 1 Department of Gastroenterology and Hepatology, Kindai University Faculty of Medicine, 377-2 Ohno-Higashi, Osaka-Sayama, 589-8511 Japan; 2 Department of Gastroenterology and Hepatology, Musashino Red Cross Hospital, Tokyo, Japan; 3 Department of Internal Medicine, Japanese Red Cross Himeji Hospital, Himeji, Japan; 4 Division of Gastroenterology and Hepatology, Department of Internal Medicine, Iwate Medical University, Iwate, Japan; 5 Department of General Surgery, Shuuwa General Hospital, Saitama, Japan; 6 Gastroenterology Center, Ehime Prefectural Central Hospital, Matsuyama, Japan; 7 Department of Gastroenterology and Oncology, Tokushima University Graduate School of Biomedical Sciences, Tokushima, Japan; 8 Department of Gastroenterology and Neurology, Kagawa University, Kagawa, Japan; 9 Department of Gastroenterology, NHO Takasaki General Medical Center, Takasaki, Japan; 10 Department of Clinical Research, NHO Takasaki General Medical Center, Takasaki, Japan; 11 Department of Gastroenterology and Hepatology, Takamatsu Red Cross Hospital, Takamatsu, Japan; 12 Department of Gastroenterology, Gunma Saiseikai Maebashi Hospital, Maebashi, Japan; 13 Department of Gastroenterology, Saiseikai Niigata Hospital, Niigata, Japan; 14 Hepatology Division, Department of Internal Medicine II, Hamamatsu University School of Medicine, Hamamatsu, Japan; 15 Department of Surgery, Kindai University Faculty of Medicine, Osaka, Japan; 16 Department of Surgery, Nagasaki University Graduate School of Biomedical Sciences, Nagasaki, Japan; 17 Department of Nursing, Gifu Kyoritsu University, Ogaki, Japan

**Keywords:** Carcinoma, Hepatocellular [MH], Immune checkpoint inhibitors [MH], Treatment outcome [MH], Conversion therapy, Drug-off

## Abstract

**Background:**

Achieving complete response (CR) is a desirable goal in early-to-intermediate-stage hepatocellular carcinoma (HCC). While systemic and locoregional therapies show promise, optimal drug discontinuation criteria remain unclear. This study aims to investigate drug-off criteria for atezolizumab plus bevacizumab as a proof-of-concept study.

**Methods:**

This retrospective multicenter study included child–pugh class A patients with unresectable HCC without extrahepatic spread or macrovascular invasion who received atezolizumab plus bevacizumab as first-line therapy. Modified clinical CR (mCCR) was defined as CR per mRECIST with sustained normal alpha-fetoprotein (AFP) levels (< 10.0 ng/dl). Recurrence-free survival (RFS) and overall survival (OS) were analyzed based on the “drug-off” criteria defined by following: (1) mRECIST CR with locoregional therapies, (2) sustained normalization of AFP/AFP-L3/ des-gamma-carboxy prothrombin (DCP) for 12–24 weeks, and (3) complete tumor vascularity disappearance by contrast-enhanced ultrasonography (CEUS) or pathological curative resection.

**Results:**

The median follow-up was 16.5 months (95% CI 15.2–17.8). Among 51 patients achieving mCCR, 11 underwent surgery, with pathological CR in three cases. In contrast, viable lesions were observed in 7 of 40 cases assessed using CEUS. All patients meeting the drug-off criteria (*n* = 9) showed no recurrence and none of them experienced mortality, while 45.2% (19/42) of those not meeting the criteria experienced recurrence (median RFS: 12.8 months, *p* = 0.007). The median OS was not reached in dug-off criteria met patients (*n* = 9), 37.7 months (95% CI: NA) in non-criteria met patients (*n* = 42), and 27.1 months (95% CI 16.7–37.6) in non-mCCR patients (*n* = 184) (*p* < 0.001).

**Conclusion:**

In patients with unresectable and TACE-unsuitable early-to-intermediate-stage HCC who met the drug-off criteria, significantly improved RFS and OS were observed compared those who did not meet the criteria. However, further validation studies are required to confirm the utility of the criteria.

## Introduction

Hepatocellular carcinoma (HCC) is a malignant neoplasm that becomes resistant to treatment as the number and size of tumors increase, as well as in the presence of vascular invasion and extrahepatic spread. Therefore, it is difficult to achieve curative outcomes, even with intensive systemic therapy, in patients with advanced-stage tumors. For HCC confined to the liver, liver transplantation, surgical resection, and radiofrequency ablation (RFA) have demonstrated high curative rates with favorable outcomes [1, 2]. However, in patients with HCC with large tumor volumes, microscopic vascular invasion, multinodular confluency, and poorly differentiated types, achieving a complete curative state becomes challenging, even in Barcelona Clinic Liver Cancer (BCLC) stage A [3, 4].

In contrast, intermediate-stage HCC (BCLC stage B) includes heterogeneous tumors with various distributions, ranging from a solitary large tumor occupying one lobe to numerous small nodules scattered throughout the liver [5]. Because of the heterogeneity of tumor status in intermediate-stage HCC, selecting curative treatments such as RFA and surgery is generally challenging, and transarterial chemoembolization (TACE) has been recommended as the standard of care. However, in recent years, the concepts of TACE failure/refractoriness [6, 7] or unsuitability [8] have been reported, leading to a transition in treatment strategies favoring upfront systemic therapy followed by TACE in patients with preserved liver function and a high tumor burden [9–13]. In the systemic therapy of unresectable, unablable, and TACE-unsuitable HCC, the combination therapy of atezolizumab plus bevacizumab is recommended as the first-line treatment based on its efficacy and tolerability [14–16]. In patients in whom bevacizumab is not suitable, durvalumab plus tremelimumab therapy is indicated [17–20]. Conventional molecular targeted agents (MTAs) such as lenvatinib or sorafenib are the first-choice treatment when combination immunotherapy is inappropriate. For the treatment of intermediate-stage HCC, it is critical to enhance treatment efficacy by considering the preservation of liver function and the emergence of adverse events. Therefore, curative conversion therapy should be considered when an anti-tumor response is obtained, because a favorable prognosis can be expected after achieving a cancer-free status.

Immunotherapy has shown good synergistic effects with locoregional therapies such as TACE and RFA/microwave ablation (MWA) [21]. The efficacy of this combination therapy is assessed using the definition of “clinical complete response (CR)” [22], which is characterized by (1) achieving CR according to mRECIST by contrast-enhanced computed tomography (CECT)/Gadolinium ethoxybenzyl diethylenetriamine pentaacetic acid (Gd-EOB-DTPA)-enhanced magnetic resonance imaging (MRI) and (2) a reduction in three tumor markers—alpha-fetoprotein (AFP), AFP-L3 fraction, and des-gamma-carboxy prothrombin (DCP)—within normal range for at least 6 weeks. This diagnostic criterion is applicable to lesions with lipiodol deposition and provides a standard for a CR. However, this is not applicable in countries where only AFP levels can be measured. Additionally, although the criteria for discontinuing immunotherapy in patients who have achieved a cancer-free state have been proposed [22, 23], their utility has not been fully validated. In this study, we retrospectively evaluated the recurrence rates after modified clinical CR (mCCR) in unresectable early-to-intermediate-stage HCC patients following curative conversion therapy, referencing “drug-off criteria” based on serum tumor markers, CECT/Gd-EOB-DTPA-enhanced MRI plus contrast-enhanced ultrasonography (CEUS) [22].

## Materials and methods

### Patients

This multicenter retrospective cohort study included 266 patients with unresectable, unablatable, and TACE unsuitable HCC of early-to-intermediate-stage from 14 medical research centers in Japan. The diagnostic criteria for HCC were determined based on histological or radiological findings following the guidelines proposed by the American Association for the Study of Liver Diseases (AASLD) [24]. Patients with Child–Pugh class A liver function were consecutively enrolled between December 1, 2021, and December 31, 2022. Follow-up regarding prognosis and recurrence was conducted with a data cutoff date of May 15, 2023.

The inclusion criteria were as follows: unresectable HCC without vascular invasion or extrahepatic spread deemed unsuitable for TACE [10]. This study included patients with BCLC stage B disease as well as those with BCLC stage A tumors that were multinodular, confluent, ^18^F-fluorodeoxyglucose (FDG) positron emission tomography (PET)-positive, or located near major vessels, which are associated with a high recurrence rate following surgical resection or ablation. Even in cases of BCLC stage A, some patients were included who were considered unresectable due to concerns of a large resectable volume, either because the tumor spanned both lobes or was located near the inferior vena cava, or due to factors such as adhesions. Eligible participants were adults aged 18 years or older with an Eastern Cooperative Oncology Group Performance Status (ECOG PS) of 0–1 and Child–Pugh class A liver function. This study included only patients who received first-line atezolizumab plus bevacizumab treatment between May 2018 and September 2022. The exclusion criteria were as follows: patients with a history of systemic therapy for HCC or other malignant tumors. Patients unable to undergo tumor evaluation using CECT or Gd-EOB-DTPA-enhanced MRI due to allergies, renal insufficiency, bronchial asthma, or other contraindications. Patients with esophageal or gastric varices at high risk of rupture. Patients with an expected survival of less than 3 months. Patients deemed unsuitable for the study by the attending physicians.

This study was conducted in compliance with the principles of the Declaration of Helsinki and was approved by the Medical Ethics Committee of Kindai University Hospital (approval number R03-218). Prior to participation, written informed consent or opt-out consent was obtained from all the patients.

### Definitions for anti-tumor response and endpoint of this study

In this study, we adopted a treatment approach that combined systemic therapy with locoregional therapy to target unresectable and TACE-unsuitable HCC. When evaluating the efficacy of immunotherapy alone, we applied RECIST v1.1 criteria, following iRECIST guidelines, which require an observation period of at least 4 weeks to confirm PD. However, when locoregional therapies such as TACE or RFA were included, residual Lipiodol deposition and post-ablation scarring made it challenging for conventional criteria, including RECIST v1.1, WHO, mRECIST, and EASL, to accurately assess pathological complete necrosis. To address this limitation, we introduced a new definition called “modified clinical complete response (mCCR).” This new mCCR definition simplifies the previously reported clinical complete response (clinical CR) criteria [22] and includes the achievement of CR based on mRECIST criteria evaluated using CECT or Gd-EOB-DTPA-enhanced MRI, along with sustained normalization of AFP levels within the normal range (< 10 ng/dL) for at least 6 weeks [25–27].

Drug discontinuation was performed in patients who underwent conversion therapy during atezolizumab plus bevacizumab treatment and achieved mCCR. In this study, we identified three criteria considered optimal for discontinuing atezolizumab plus bevacizumab, which we named the “drug-off criteria”. These criteria include: (1) achieving mRECIST CR through superselective TACE/RFA/MWA, (2) sustained normalization of AFP/AFP-L3/DCP for 12–24 weeks or longer, and (3) complete disappearance of tumor vascularity, as evaluated by CEUS or pathologically confirmed curative resection [22]. Even in cases with all baseline tumor markers negative, it was required to maintain negative tumor markers during treatment.

The primary endpoint of this study was the recurrence-free survival (RFS) in patients who achieved mCCR and met the drug-off criteria. Secondary endpoints included the objective response rate (ORR) and disease control rate (DCR) per RECISTv1.1 of atezolizumab plus bevacizumab therapy, the rate of achieving mCCR after locoregional therapy, the rate of achieving the drug-off criteria, and overall survival (OS).

### Treatment strategy for atezolizumab plus bevacizumab curative conversion therapy

**Fig. 1:**
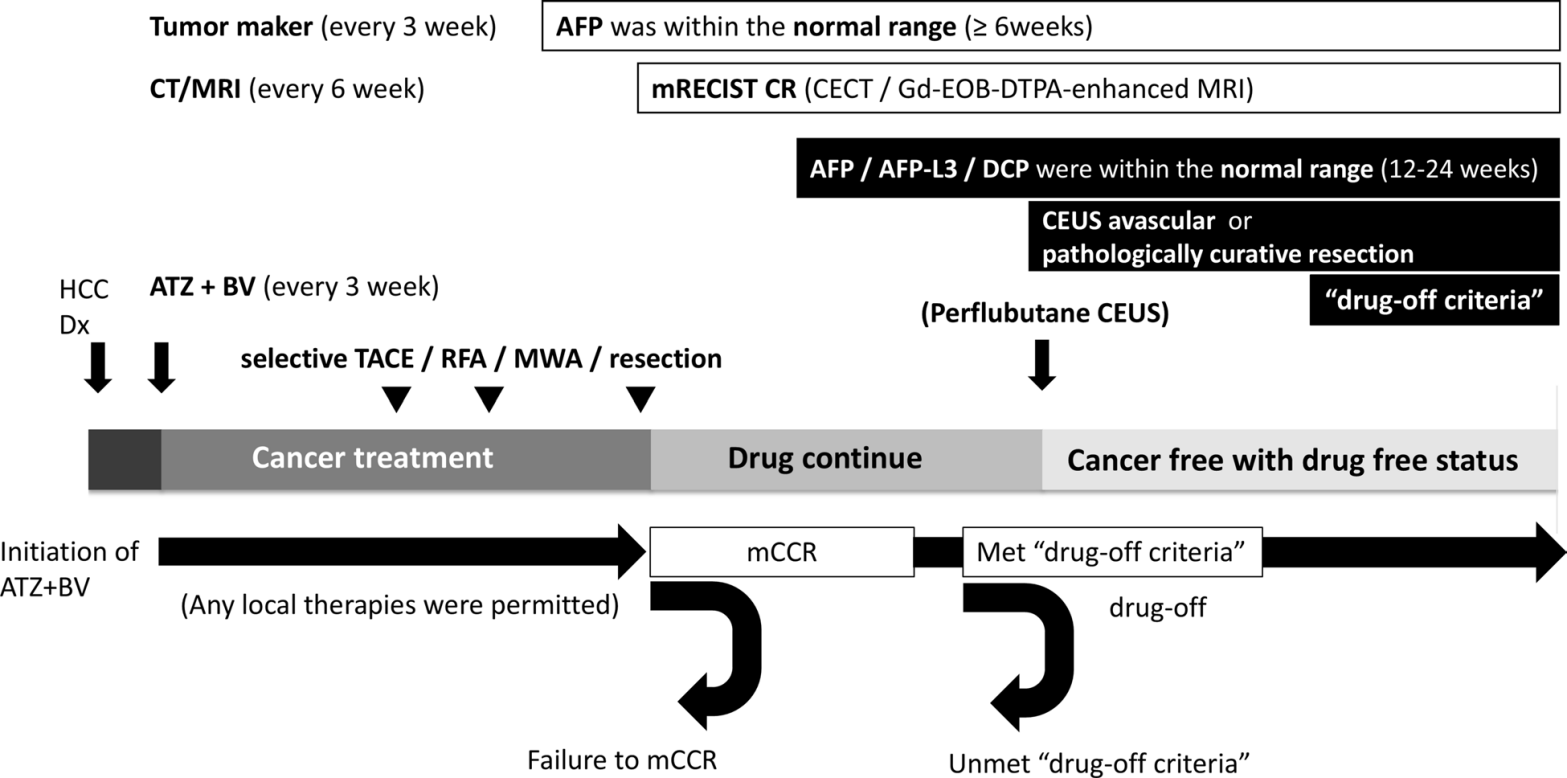
Treatment protocol of atezolizumab plus bevacizumab curative conversion therapy. Patients diagnosed with unresectable and transarterial chemoembolization (TACE)-unsuitable hepatocellular carcinoma (HCC) were treated with atezolizumab plus bevacizumab every 3 weeks. Serum tumor marker assessments were performed every 3 weeks, and imaging evaluations with contrast-enhanced computed tomography/magnetic resonance imaging were conducted every 6 weeks. Optional locoregional therapies were added after 3–4 cycles of drug administration. “Modified clinical complete response (mCCR)” was defined as maintaining alpha-fetoprotein (AFP) levels within the normal range for ≥ 6 weeks and achieving CR according to modified Response Evaluation Criteria in Solid Tumors (mRECIST). Subsequently, “drug-off criteria” were met if AFP/AFP-L3 fraction/des-gamma-carboxy prothrombin (DCP) remained within the normal range for 12–24 weeks and if pathological cure was confirmed through surgical resection or tumor vascularity disappeared on contrast-enhanced ultrasound (CEUS)

The treatment protocol used in this study is shown in Fig. 1. Atezolizumab plus bevacizumab combination therapy was administered every 3 weeks to patients with unresectable and TACE-unsuitable HCC. Each attending physician assessed the anti-tumor effect on the target lesion based on RECIST v1.1 [28]. Dose reductions or interruptions were considered if the patients presented severe adverse events defined in the Common Terminology Criteria for Adverse Events (CTCAE) v4 (Grade ≥ 3) [29, 30]. After 3–4 cycles, the response to the combination therapy was evaluated to determine the indications for additional locoregional therapy. For patients who achieved a partial response (PR) or CR according to RECIST v1.1, using atezolizumab plus bevacizumab, aggressive conversion therapy, including attempted curative RFA/MWA/surgical resection, was selected. In cases where the treatment response was stable disease (SD) according to RECIST v1.1, lenvatinib-TACE therapy, followed by continuation of atezolizumab plus bevacizumab therapies, was administered. In cases of progressive disease (PD), according to RECIST v1.1 or when treatment was discontinued due to adverse events, superselective TACE procedures were performed before treatment discontinuation. Atezolizumab plus bevacizumab were resumed when possible, and locoregional therapy was administered on-demand.

During systemic therapy, tumor markers were measured every 3 weeks, and CECT or Gd-EOB-DTPA-enhanced MRI scans were performed every 6 weeks. If serum AFP levels remained negative for at least 6 weeks and CR was confirmed by the mRECIST assessment based on CECT/Gd-EOB-DTPA-enhanced MRI, it was determined to be mCCR. After achieving mCCR, tumor marker monitoring and imaging examinations were conducted, and tumor vascularity was assessed using perflubutane CEUS to evaluate viable lesions. Atezolizumab plus bevacizumab were continued after achieving mCCR, and the decision regarding drug discontinuation and timing was left to the discretion of the attending physician at each site.

### Clinical and laboratory evaluation

The following clinical examinations were conducted within 4 weeks prior to the initiation of atezolizumab plus bevacizumab therapy: body weight, height, body mass index (BMI), body surface area (BSA), white blood cell count, neutrophil-to-lymphocyte ratio (NLR), platelet count, prothrombin time-international normalized ratio (PT-INR), serum albumin, total bilirubin, aspartate aminotransferase (AST), alanine aminotransferase (ALT), thyroid function, general urinalysis, AFP, AFP-L3 fraction, and DCP. The albumin-bilirubin (ALBI) score was calculated using the following formula: (log10 [total bilirubin (mg/dL) × 17.1] × 0.66) + (albumin [g/dL] × 10 × −0.085 [31].

### CEUS examination

For patients who achieved mCCR, we attempted to visualize all intrahepatic nodules using B-mode ultrasound scan and CEUS scan. Patients in which visualization and blood flow assessment of all intrahepatic nodules were not feasible were classified as “not detectable” on CEUS and excluded from further CEUS assessment. For patients in which all intrahepatic nodules could be evaluated, the presence or absence of tumor blood flow in the HCC nodules was visually assessed. B-mode ultrasound scans were obtained using a LOGIQ E9 (GE Healthcare, Chalfont St. Giles, UK) or an Aplio i500/i800 imaging system (Canon Medical Systems, Japan) with a convex probe. The acoustic power for contrast harmonic sonography was set to the default setting with a mechanical index of 0.2. A single focal point is set at the deepest part of the monitor. The ultrasound contrast agent used was Sonazoid™ (perflubutane microbubbles with a mean diameter of 2–3 μm; GE Healthcare, Japan). One vial of perflubutane was dissolved in 2 ml of distilled water, and a solution of 0.01 ml/kg was injected as a bolus via a 22–24 gauge intravenous catheter, followed by a 10 ml flush of normal saline. After the injection, the target lesions were scanned in the arterial and Kupffer phases. The arterial phase of CEUS was defined as occurring between 10 and 60 s after Sonazoid injection, while the Kupffer phase was defined as occurring 10 min after injection [32, 33].

### Statistical analyses

Statistical analyses were performed using the IBM Statistical Package for the Social Sciences (version 22.0; IBM, Armonk, NY, USA) and R version 4.3.0 (https://cran.r-project.org/bin/windows/base/). Statistical significance was set at *p* < 0.05. For the analysis of clinical characteristics, chi-square and Fisher’s exact tests were conducted to ensure that the expected values were greater than five for the chi-square tests. Continuous variables are presented as median [range or interquartile range (IQR)] as appropriate, and analysis of variance, one-way analysis of variance, and Mann–Whitney *U* tests were performed. Kaplan–Meier analysis with the log-rank test was conducted using SPSS and the R packages survival and survminer to compare OS and RFS. Additionally, R packages ggalluvial, tidyverse, and stringr were utilized for visualization and data manipulation.

## Results

### Patient characteristics

Of the 266 enrolled patients, 235 met the inclusion criteria and were analyzed in this study. The data cutoff date was May 15, 2023, and the median follow-up duration was 16.5 months [95% confidence interval (CI) 15.2–17.8]. The details of the 235 patients are presented in Table 1. The median age was 74.0 years old (range 41–94), with males accounting for 74.0% (174/235) of the participants. Hepatitis B surface antigen (HBsAg) was positive in 12.8% (30/235) of cases, whereas hepatitis C virus antibody (HCVAb) was observed in 36.2% (85/235) of cases. The percentage of patients who tested negative for both HBs antigen and HCV antibodies was 51.1% (120/235). A total of 158 cases had a history of locoregional therapies such as liver resection, RFA, and TACE, but none of the cases had received systemic therapy. The median maximum diameter of the tumor was 3.4 cm (range 0.7–20.0), and 135 cases had four or more intrahepatic HCC nodules. The median baseline AFP level was 22.6 ng/dL (range 1–238,000), with a median AFP-L3 fraction of 7.0% (range 0.5–95.2). The median DCP level was 200.5 mAU/mL (range 1–365,759). The study included eight cases with all negative baseline levels of AFP, DCP, and AFP-L3 fractions.

**Table 1:** Baseline characteristic of patients with unresectable and TACE unsuitable HCC

Factors	Unit categories	All patients (n = 235)	Achieved “mCCR”^a^ (n = 51)	Failure to “mCCR”^a^ (n = 184)	*p* value
Age	Years old, median (range)	74.0 (41–94)	76.0 (51–94)	74.0 (41–92)	0.193
Sex	Male/female	174/61	41/10	133/51	0.282
PS	0/1	200/35	39/12	161/23	0.061
BMI	kg/m^2^, median (range)	23.8 (15.7–37.6)	24.6 (18.4–34.1)	23.6 (15.7–37.6)	0.505
Etiology	HBV/HCV/AL/NAFL/other	30/85/56/48/16	4/20/14/12/1	26/65/42/36/15	0.047
Prior locoregional therapy	Yes/No	158/77	25/26	133/51	< 0.01
BCLC stage	A/B up-to-7 IN/B OUT	33/49/153	14/7/30	19/42/123	0.011
Tumor size	cm, median (range)	3.4 (0.7–20.0)	3.8 (1.0–14.7)	3.2 (0.7–20.0)	0.038
Tumor number	1–3/4–6/ ≥ 7	100/81/54	31/13/7	69/68/47	0.006
Child–Pugh score	5/6	166/69	42/9	124/60	0.094
ALBI score	Median (IQR)	− 2.56 (− 2.87, − 2.23)	− 2.67 (− 2.90, − 2.34)	− 2.53 (− 2.87, − 2.22)	0.397
NLR	Median (IQR)	2.3 (1.5, 3.4)	2.0 (1.4, 3.1)	2.5 (1.7, 3.4)	0.109
PLT	Median (IQR)	14.0 (10.9, 18.6)	14.4 (12.8, 19.2)	13.9 (10.6, 18.5)	0.451
PT-INR	Median (IQR)	1.08 (1.00, 1.14)	1.07 (1.00, 1.14)	1.1 (1.00, 1.12)	0.421
ALB	Median (IQR)	3.9 (3.5, 4.2)	4.0 (3.7, 4.3)	3.9 (3.5, 4.2)	0.267
T-bil	Median (IQR)	0.8 (0.6, 1.0)	0.8 (0.6, 1.0)	0.8 (0.6, 1.0)	0.995
CRP	Median (IQR)	0.18 (0.09, 0.44)	0.18 (0.1, 0.44)	0.28 (0.08, 0.42)	0.921
ALT	Median (IQR)	31 (20, 47)	27 (18, 47)	31 (21, 42)	0.626
AFP	Median (range)	22.6 (1–238000)	10.0 (1–238000)	30.2 (2–100000)	0.439
AFP-L3 fractions	Median (range)	7.0 (0.5–95.2)	4.5 (0.5–91.3)	9.0 (0.5–95.2)	0.016
DCP	Median (range)	200.5 (1–365759)	316.1 (13–247031)	188.3 (1–365759)	0.625
All negative levels of AFP, DCP, and AFP-L3 fractions	Yes/No	8/227	4/47	4/180	0.100

Values were express in median (range)

*PS* performance status, *BMI* body mass index, *HCC* hepatocellular carcinoma, *HCV* hepatitis C virus, *HBV* hepatitis B virus, *AL* alcohol induced hepatitis, *NAFL* nonalcoholic fatty liver, *BCLC* Barcelona Clinic Liver Cancer, *NLR* Neutrophil–Lymphocyte Ratio, *CRP* C-reactive protein, *ALBI* albumin-bilirubin, *ALT* alanine aminotransferase, *AFP* α-fetoprotein, *AFP-L3* Lectin-reactive fraction of alpha-fetoprotein, *DCP* des-γ-carboxy prothrombin, *IQR* interquartile range, *mRECIST* modified Response Evaluation Criteria in Solid Tumors, *CR* complete response

^a^“mCCR(modified clinical CR)” was defined by mRECIST CR and AFP levels within the normal range (< 10.0 ng/dL)

**Fig. 2:**
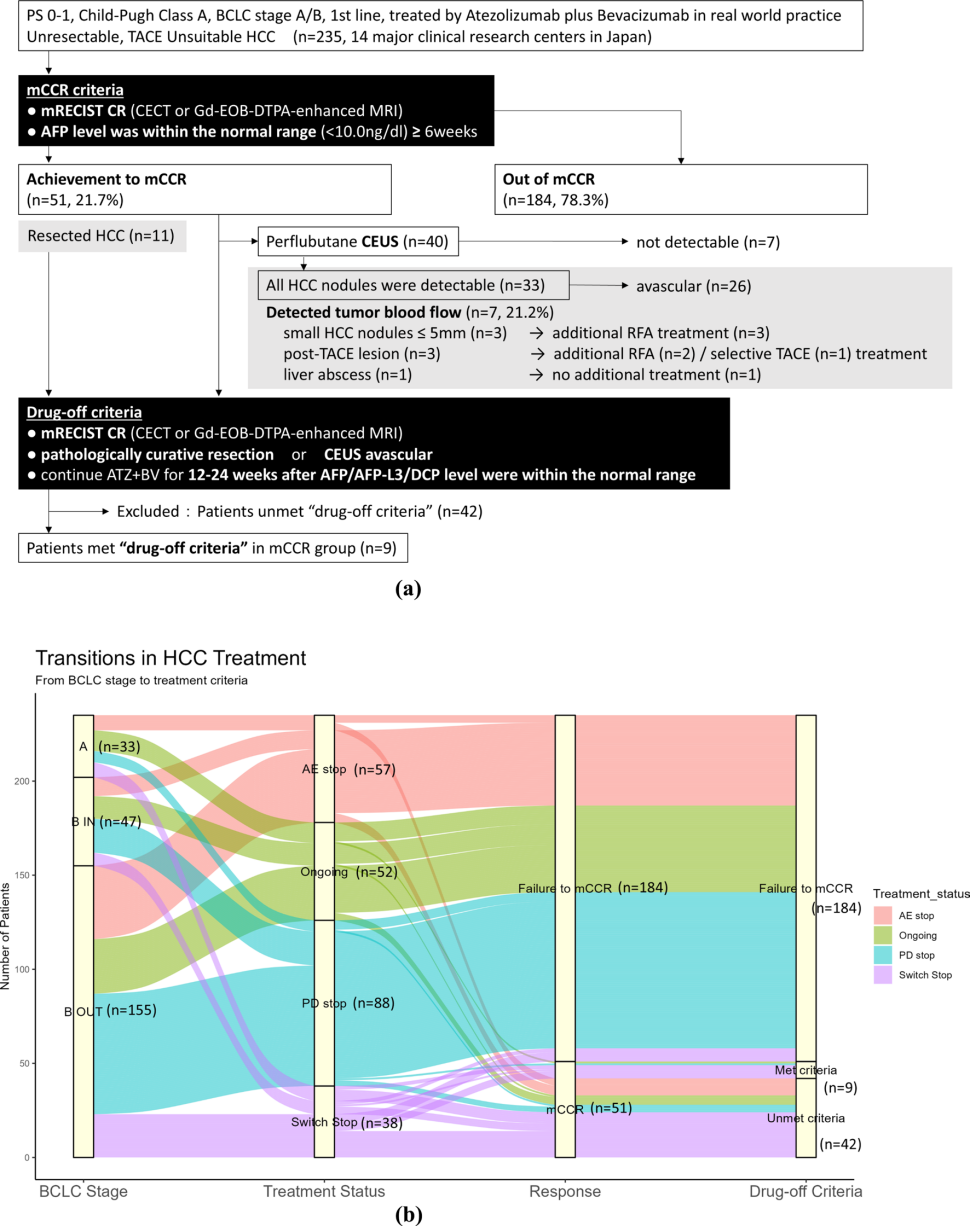
**a** Schematic of prospective cohort study design. A total of 235 patients who met the inclusion criteria were enrolled in this study. Among them, 51 achieved mCCR, and of these, 11 were confirmed to have achieved curative pathological resection. Among the remaining 40 patients, perflubutane contrast-enhanced ultrasound (CEUS) was performed in 33 patients, and subtle tumor vascularity was observed in 7 patients, leading to the achievement of curative conversion through additional locoregional therapy. Ultimately, 9 patients met the “drug-off criteria.” **b** Transitions in ABC conversion treatment. In patients with BCLC Stage A/B unresectable, TACE-unsuitable, or TACE-refractory HCC treated with atezolizumab plus bevacizumab, at the time of discontinuation of observation, 52 patients were still on treatment, 38 patients discontinued due to transition to other locoregional therapies, 57 patients discontinued due to adverse events (AEs), and 88 patients discontinued due to disease progression (PD). While most patients who discontinued treatment due to AEs, PD, or were continuing on the drug did not achieve mCCR, those who discontinued atezolizumab plus bevacizumab to transition to other therapies were able to achieve mCCR. Of the 51 patients who achieved mCCR, 9 met the drug-off criteria

According to the protocol, a combination therapy of atezolizumab plus bevacizumab was administered along with conversion therapies. Among the 235 patients, 11 underwent conversion therapy with surgical resection, 13 underwent RFA/MWA, and 38 underwent selective TACE (+ RFA/MWA). Among them, mCCR was achieved in 48 patients. Additionally, three patients met the criteria for mCCR solely with atezolizumab plus bevacizumab therapy without any additional locoregional treatment. Consequently, 51 patients (21.7%; 51/235) achieved mCCR, whereas the remaining 184 patients (78.3%) were unable to achieve mCCR (Fig. 2a).

Characteristics of mCCR achievers and non-achievers are summarized in Table 1. There was a significantly higher proportion of HBsAg-positive cases among patients who failed to achieve mCCR than among those with mCCR (mCCR vs. non-mCCR: 7.8 vs. 14.1%, *p* = 0.047). Additionally, significant differences were observed in baseline maximum diameter of the tumor between patients with mCCR and non-mCCR (3.8 vs. 3.2 cm, *p* = 0.038) and in number of intrahepatic nodules (median number of tumors 3 vs. 5, *p* = 0.011). The BCLC stage A HCC category included patients with a combination of factors, such as confluent multinodular tumors, microvascular invasion, and poorly differentiated HCC with uptake of ^18^F-FDG accumulation on PET-CT. These conditions make it challenging to achieve complete remission using locoregional therapy alone. Baseline AFP level and DCP level were not significantly different between patients who achieved mCCR and those who failed to achieve mCCR; the AFP-L3 fraction was significantly higher in patients without mCCR (mCCR vs. non-mCCR: 4.5 vs. 9.0%, *p* = 0.016).

### Efficacy and safety of atezolizumab plus bevacizumab therapy and curative conversion treatment

The tumor responses to atezolizumab plus bevacizumab therapy in this cohort are presented in Table 2a. Among the 235 patients, 6 (2.6%) achieved CR, 74 had a partial response (PR), 106 had stable disease (SD), and 38 had progressive disease (PD) according to RECIST v1.1 criteria. The objective response and disease control rates (ORR) was 34.0% (80/235) and the disease control rate (DCR) was 79.1% (186/235), respectively. Among the 6 patients with CR, 1 achieved mCCR without additional curative locoregional treatment. The remaining five patients did not meet the criteria for mCCR due to sustained elevation of AFP levels or PD in other lesions during atezolizumab plus bevacizumab treatment. Among the 74 patients classified as having a PR, 33 achieved mCCR. Of these, 2 patients achieved mCCR without conversion treatment, 7 underwent surgical resection, and 3 showed pathological complete necrosis. Among the 24 patients classified as PR, mCCR was achieved using TACE or RFA/MWA. Among the 106 cases classified as SD, 14 achieved mCCR, of which four underwent conversion surgical resection, with pathologically viable lesions observed in all cases. In 10 patients classified as having SD, mCCR was achieved using TACE or RFA/MWA. Among the 38 cases classified as PD, 3 patients achieved mCCR with additional TACE at the time of discontinuation of atezolizumab plus bevacizumab therapy due to adverse events or tumor progression (Table 2a). The reasons for continuation or discontinuation of atezolizumab plus bevacizumab therapy, including discontinuation due to AE, PD, or transition to other treatments, are summarized in Fig. 2b for the 235 patients analyzed. The figure visualizes the distribution of cases by BCLC stage, highlighting which patients discontinued atezolizumab plus bevacizumab and the proportion of patients who achieved mCCR among those continuing therapy. This analysis reveals that most patients who continued therapy or discontinued due to AE or PD failed to achieve mCCR. In contrast, patients who discontinued therapy to transition to other treatments showed a higher proportion of mCCR achievement. Additionally, among patients who achieved mCCR, those who discontinued therapy due to AE or continued drug therapy were less likely to meet the drug-off criteria. The AEs observed with atezolizumab plus bevacizumab therapy did not exceed those previously reported [14]. No major adverse events were encountered when combined with conversion therapy, apart from complications such as bile leakage.

**Table 2:** (a) Objective response rate (ORR) and disease control rate (DCR) of atezolizumab plus bevacizumab therapy per RECIST v1.1

Response category (RECISTv1.1)	All pts (n = 235)	Pts who achieved mCCR (n = 51)
CR, number	6	1
PR, number	74^a^	33
SD, number	106	14
PD, number	38	3
NE, number	11	0
ORR, %	34.0% (80/235)	–
DCR, %	79.1% (186/235)	–

(b) Number of cases achieving “radiological complete response (CR)” and “drug-off criteria” in each conversion therapy category for unresectable and TACE-unsuitable Hepatocellular carcinoma		
Conversion therapy	Achieved mCCR (n = 51)	Patients met drug-off criteria (n = 9)
Surgical resection (+ selective TACE)	11 (21.6%)	4 (44.4%)
RFA / MWA	11 (21.6%)	3 (33.3%)
TACE or lenvatinib-TACE sequential (+ RFA/MWA)	26 (50.9%)	2 (22.3%)
No additional treatment (ATZ + BV only)	3 (5.9%)	0 (0.0%)

*RECISTv1.1* Response Evaluation Criteria in Solid Tumors version 1.1, *CR* complete response, *PR* partial response, *SD* stable disease, *PD* progressive disease, *NE* not evaluated, *ORR* objective response rate, *DCR* disease control rate, *mCCR* modified clinical CR, *MWA* microwave ablation, *RFA* radiofrequency ablation, *TACE* transarterial chemoembolization, *ATZ + BV* atezolizumab plus bevacizumab, *mCCR* modified clinical complete response

^a^Pathological complete necrosis was achieved in 3 out of 74 cases with PR

### Achievement of drug-off criteria with perflubutane CEUS examination

Of the 51 patients who achieved mCCR, 11 underwent surgical resection, and among the remaining 40 patients, the assessment of the vascularity of HCC nodules using perflubutane CEUS examination was feasible in 33 patients (Fig. 2a). Among these 33 cases, seven (21.2%) revealed hypervascular tumors on CEUS, which were originally diagnosed as hypovascular by CECT or Gd-EOB-DTPA-enhanced MRI. Three of the seven patients had small intrahepatic metastases less than 5 mm in diameter, and subsequent RFA treatment led to a CR with the disappearance of vascularity. In the other three cases with lipiodol deposition after TACE, additional RFA (*n* = 2) and superselective TACE (*n* = 1) were performed on the viable lesions, leading to a CR. The remaining patient had a liver abscess caused by TACE, and significant tumor shrinkage was achieved during observation. Collectively, among the 51 patients who achieved mCCR, 9 cases (17.6%) met the "drug-off criteria,” which included cases with pathological CR, disappearance of tumor perfusion confirmed by CEUS, and normalization of serum AFP, AFP-L3 fraction, and DCP levels for 12–24 weeks (Fig. 2a). Among the nine patients that met the drug-off criteria, eight discontinued atezolizumab plus bevacizumab after a median of 28 weeks (range 12–137 weeks) of continued treatment following the normalization of tumor markers. One case has been receiving atezolizumab plus bevacizumab for over 63 weeks and remains on treatment at the end of the observation period. The numbers of patients who achieved mCCR or met the drug-off criteria for each conversion therapy are summarized in Table 2b.

**Fig. 3:**
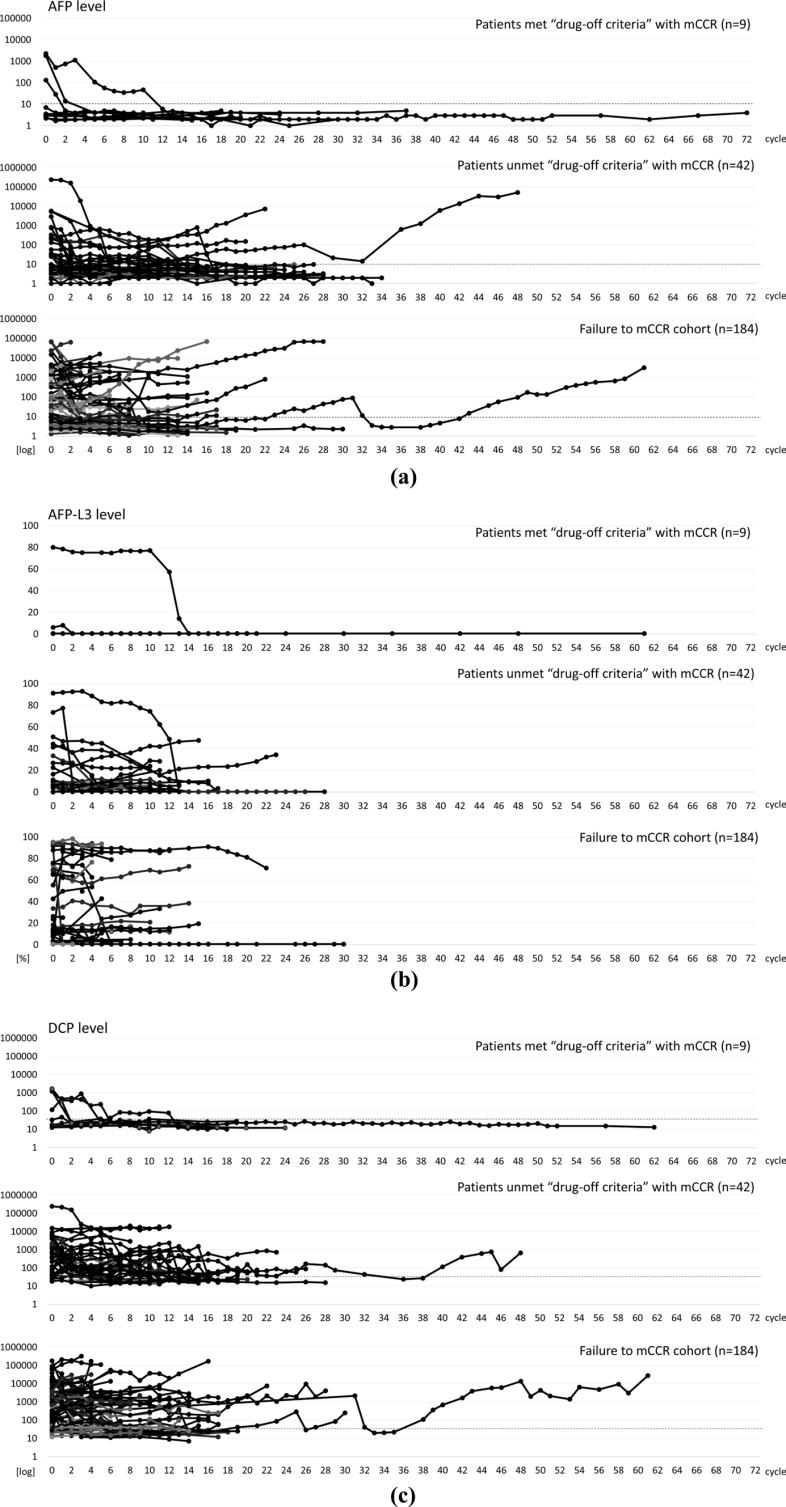
Spider plots of serum tumor marker. Serum tumor markers after initiation of atezolizumab plus bevacizumab combination therapy presented as spider plots divided into three groups: **a** AFP levels, **b** AFP-L3 fraction, and **c** DCP levels

Subsequently, we categorized the patients into three groups: those meeting the drug-off criteria and achieving mCCR (*n* = 9), those achieving mCCR but not meeting the drug-off criteria (*n* = 42), and those who failed to achieve mCCR (*n* = 184). Spider plots of each tumor marker were created for the three groups (Fig. 3a–c). It was demonstrated that even in patients achieving mCCR, a considerable number of patients experienced an increase in serum AFP, AFP-L3 fraction, or DCP levels.

### Recurrence-free survival

**Fig. 4:**
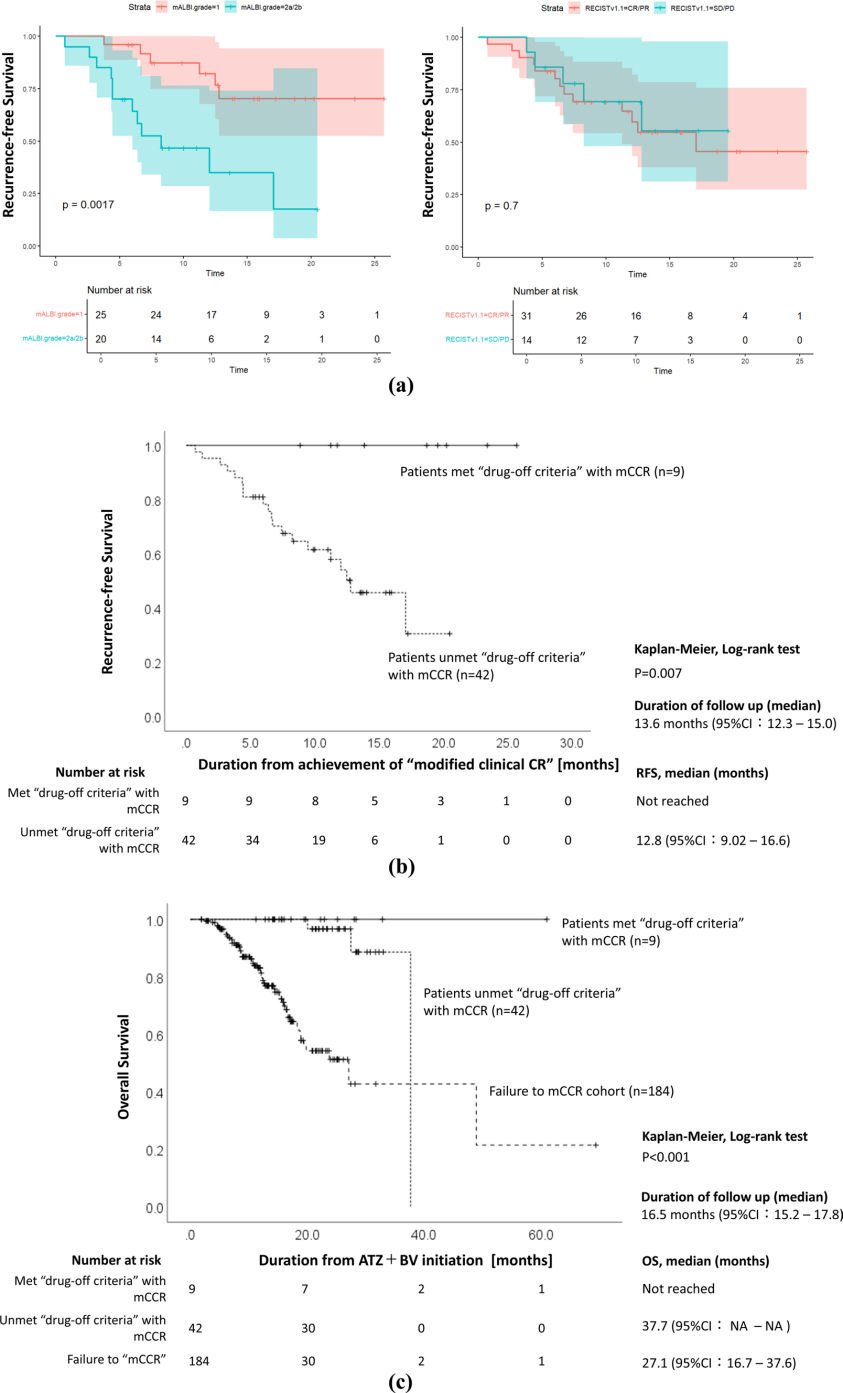
Kaplan–Meier curve for recurrence-free survival and overall survival. We investigated factors contributing to the recurrence-free survival (RFS) after achieving mCCR, and the results are presented in (**a**) and (**b**). **a** RFS according to mALBI grade and the response to atezolizumab plus bevacizumab based on RECIST v1.1 are shown. **b** RFS between patients who met the drug-off criteria and those who did not. Among the patients who met the “drug-off criteria” (*n* = 9), none experienced recurrence, while among those who did not meet the drug-off criteria with mCCR (*n* = 42), 19 patients showed recurrence. **c** overall survival (OS) analysis was performed on the 235 patients. Patients who did not achieve mCCR had significantly worse prognosis, whereas those who met the drug-off criteria had the most favorable prognosis

We investigated factors contributing to recurrence in 51 patients who achieved mCCR. The median follow-up period for RFS was 13.6 months (95% CI 12.3–15.0). The results of the univariate Cox proportional hazards regression analysis for recurrence after achieving mCCR are presented in Table 3. Among the examined factors, only mALBI grade 2a/2b showed a statistically significant association with recurrence. Next, factors contributing to recurrence were evaluated using Kaplan–Meier analysis with the log-rank test. The results are shown in Fig. 4a and b. Drug response to atezolizumab plus bevacizumab, as assessed by RECIST v1.1, had no significant impact on RFS. Patients with mALBI grade 2a/2b had significantly poorer RFS compared to those with mALBI grade 1.

**Table 3:** Univariate Cox proportional hazards regression analysis for recurrence after achieving mCCR

	HR	95% CI (lower)	95% CI (upper)	*p* value
Age	0.990	0.93	1.05	0.749
Sex	0.467	0.16	1.34	0.157
PS	0.537	0.15	1.86	0.327
mALBI grade2a/2b	4.390	1.61	12.0	0.004
RECISTv1.1 SD/PD	0.820	0.29	2.29	0.702
BCLC stage B OUT	0.689	0.23	2.04	0.502
NLR	0.951	0.69	1.31	0.756
PLT	1.013	0.92	1.11	0.787
AFP	0.999	1	1	0.642
DCP	0.999	1	1	0.568
AFP-L3	1.002	0.99	1.02	0.852

In the analysis of RECISTv1.1, CR and PR were combined into a single category and used as the baseline for calculating hazard ratios (HRs)

Among the patients who met the “drug-off criteria” with mCCR (*n* = 9), eight patients successfully discontinued the atezolizumab plus bevacizumab treatment, while one patient is still receiving the combination therapy for over 137 weeks (Fig. 2b). None of the patients experienced HCC recurrence during the observation period (median RFS was not reached) (Fig. 4b). In contrast, among patients who unmeet the “drug-off criteria” with mCCR (*n* = 42), atezolizumab plus bevacizumab combination therapy was discontinued in 28 cases due to adverse events, patient preference, or physician’s judgment. Among them, 45.2% (19/42) experienced HCC recurrence. The median RFS was 12.8 months (95% CI 9.02–16.6), and the recurrence rate was significantly higher compared to those who met the drug-off criteria with mCCR (log-rank test, *p* = 0.007) (Fig. 4b). The patterns of recurrence were as follows: six cases showed local recurrence of the targeted tumor at the initiation of atezolizumab plus bevacizumab combination therapy, 13 cases showed metachronous recurrence after curative conversion therapy. Among the patients with recurrence, tumor progression was controlled in ten patients by RFA/MWA or TACE, while nine cases required the resumption of systemic therapy or on-demand TACE due to the failure of curative locoregional therapy.

### Overall survival

OS was assessed in 235 patients included in the study (Fig. 4c). Among patients who did not achieve mCCR (*n* = 184), the median OS was 27.1 months (95% CI 16.7–37.6). Among patients who unmeet the “drug-off criteria” with mCCR (*n* = 42), the median OS was 37.7 months (95% CI NA–NA). In contrast, all patients who met the “drug-off criteria” with mCCR (*n* = 9) were still alive, and the OS was not reached, indicating their significantly favorable prognosis (log-rank test, *p* < 0.001).

## Discussion

The treatment strategies for unresectable HCC have evolved significantly. Systemic therapies are now recommended not only for advanced HCC but also for intermediate-stage HCC in patients who are TACE-unsuitable or TACE-refractory [9–13]. In particular, patients with unresectable Intermediate-stage HCC that is TACE-unsuitable or TACE-refractory, which are typically confined to the liver, represent a group for whom the combination of systemic therapy and locoregional therapy may aim for curative outcomes. Several studies focusing exclusively on intermediate-stage HCC have reported the outcomes of combination therapies involving systemic and locoregional treatments. In phase III trials combining sorafenib with DEB-TACE, such as the SPACE trial and the TACE2 trial, the mRECIST-based CR rates were reported to be between 13 and 29%, which were comparable to those in the DEB-TACE monotherapy arms [34, 35]. Conversely, in the phase II TACTICS trial, the combination of sorafenib and TACE achieved a CR rate of 28.8% (ORR 71.3%) using mRECIST criteria [36, 37]. Similarly, in the TACTICS-L trial evaluating lenvatinib combined with TACE, the CR rate was reported as 66.1% (ORR 85.5%) using the mRECIST [38, 39]. In a retrospective clinical trial combining atezolizumab plus bevacizumab, conversion therapy was achieved in 34.5% of cases, all of which resulted in clinical CR [22].

Next, we will discuss the results in populations that include BCLC stage C. The LAUNCH trial, a multicenter phase III study comparing lenvatinib alone and lenvatinib plus on-demand TACE, reported that conversion surgery was performed in 15.3% of cases (26/170), with complete pathological necrosis observed in 1.2% (2/170) [40]. Similarly, in the phase III EMERALD-1 trial (durvalumab + bevacizumab + TACE) and the phase II trial combining Y90-radioembolization with nivolumab (CA 209–678), the CR rate per RECISTv1.1 was reported to be 3.0%. However, these trials did not examine drug discontinuation criteria or recurrence rates after discontinuation of therapy.

For systemic therapy alone, including patients with BCLC stage C, the CR rates, based on RECISTv1.1, for unresectable HCC using MTAs such as lenvatinib [41], sorafenib [42], ramucirumab [43], cabozantinib [44], and regorafenib [45], are below 1%. However, the combination immunotherapy of atezolizumab plus bevacizumab showed a CR rate of 7.7% (ORR 29.8%) [14, 46], that of the combination of durvalumab and tremelimumab was 3.1% (ORR 20.1%) [47], and that of the combination of nivolumab and ipilimumab was 7% (ORR 36%) [48].

In previous clinical trials, there have been limited investigations into drug discontinuation and recurrence following achievement of CR, indicating a scarcity of evidence in this area. In this analysis, we proposed the drug-off criteria and examined the recurrence rates when these criteria were adhered to. Patients who met the drug-off criteria showed a significantly lower recurrence rate following mCCR achievement, and OS significantly improved (Fig. 4a and b).

Patients with HCC who maintain a normal range of AFP levels and the disappearance of blood flow in the tumor on CT or MRI are generally considered to be in a necrotic state. However, in our study, we observed HCC recurrence in 45.2% of patients who achieved mCCR, although they did not meet the drug discontinuation criteria, with a median RFS of 12.8 months (95% CI 9.02–16.6). Among the cases of recurrence, 68.4% (13/19) were characterized as metachronous recurrence, whereas 52.6% achieved complete tumor control with additional locoregional therapy. These findings revealed the risk of judging complete tumor control solely by CT or MRI and AFP normalization, i.e., by the mCCR criterion. In our analysis of factors contributing to recurrence after achieving mCCR, no consistent trend was observed that linked the drug response to atezolizumab plus bevacizumab (as assessed by RECIST v1.1). However, patients who met the drug-off criteria did not experience any recurrences (as no recurrences occurred, statistical analysis could not be performed). Notably, the persistent normalization of the three tumor markers and the absence of tumor blood flow on imaging, including CEUS, appear to be crucial factors in preventing recurrence.

Globally, serum AFP level is commonly used as a tumor marker for HCC, whereas in Japan, the AFP-L3 fraction and DCP are also measured simultaneously. As shown in the spider plots in Fig. 3, there were many cases in which the AFP-L3 fraction and DCP levels were persistently elevated, even after achieving mCCR with a normal AFP level as an essential criterion. Therefore, it is important to measure all three HCC markers to define complete tumor control. Additionally, although CECT and Gd-EOB-DTPA-enhanced MRI indicated complete disappearance of tumor blood flow, CEUS with high temporal and spatial resolution could detect nodules smaller than 5 mm. Therefore, we believe that CEUS is critical to accurately assess CR, and that CECT and Gd-EOB-DTPA-MRI alone are insufficient for precise evaluation.

**Fig. 5:**
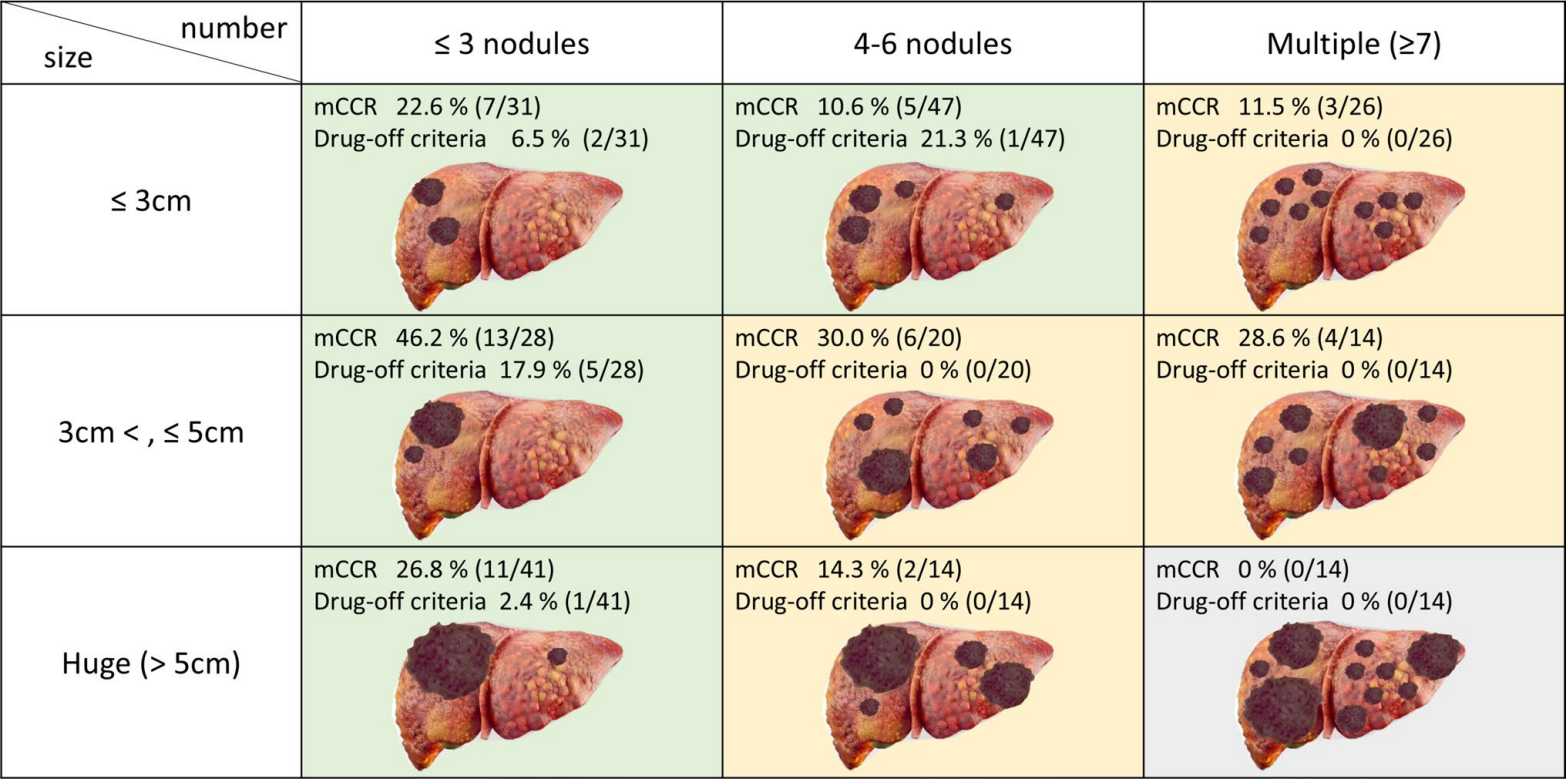
The achievement rates of mCCR and drug-off criteria based on the number of intrahepatic HCC nodules and tumor maximum diameter. Categories containing patients who met the drug-off criteria are indicated in green, whereas categories in which neither mCCR nor the drug-off criteria were achieved are shown in gray

Next, it was necessary to determine which patients met the mCCR and drug-off criteria. Figure 5 shows the percentage of patients who achieved mCCR and drug-off status according to the number of nodules and maximum tumor diameter. The results revealed that patients who met the drug-off criteria included those with three or fewer nodules, and patients with a maximum tumor diameter of 3 cm or less and six or fewer nodules (indicated in green in Fig. 5). In contrast, no patients with a tumor diameter of 5 cm or larger with more than four nodules and seven or more HCC nodules, which were considered to have a high tumor burden, achieved mCCR or met the drug-off criteria (shown in gray in Fig. 5). Based on these findings, tumor number is considered the most critical factor for tumor control through an integrative treatment approach, primarily utilizing atezolizumab plus bevacizumab. Even in cases with a large maximum tumor diameter, if the tumor number is within the range of 1–3, there is significant potential for successful curative conversion with atezolizumab plus bevacizumab plus locoregional therapy, regardless of the presence of a multinodular confluent type, poorly differentiated type, or high ^18^F-FDG uptake in HCC.

The limitations of this study are as follows: it is a retrospective cohort study conducted only in Japanese ethnic groups, the sample size of patients who met the drug discontinuation criteria was small, and the background patient characteristics of the three groups defined by the mCCR and drug discontinuation criteria were not well balanced, which may affect the comparisons of OS and RFS. In addition, while the drug-off criteria included blood flow assessment using CEUS as a mandatory component, it is important to note that in cases with multiple lesions or certain locations, complete evaluation by ultrasound is often difficult, which is a limitation of this criteria.

Furthermore, HCCs with a small number of tumors and small tumor size, even those with a high biological malignant grade, may have the potential for curative conversion with locoregional therapies, such as TACE (+ RFA/MWA), surgical resection, or heavy-ion radiation. It remains uncertain whether atezolizumab plus bevacizumab followed by curative conversion therapy truly improves RFS in these patients, as sufficient data are not currently available. Therefore, an ongoing prospective clinical trial, the IMPACT trial [49] that compares OS between atezolizumab plus bevacizumab alone and atezolizumab plus bevacizumab combined with a curative conversion approach is awaiting. This study was planned as a proof-of-concept study and retrospectively evaluated RFS based on the previously proposed drug-off criteria [22].

## Conclusion

Previously, CR was rarely expected in unresectable intermediate-stage HCC. However, the combination of systemic and locoregional therapies has shown promising results in terms of achieving cancer-free and drug-free statuses. With the advent of immunotherapy-based combination therapy with locoregional therapy, the assessment of the timing of discontinuation of systemic therapy is critical for achieving a drug-free status and favorable outcomes, i.e., long-term survival. Although the proposed criteria for drug discontinuation are still insufficiently validated, this study has clarified the significant improvement in RFS and OS even after drug discontinuation based on the proposed drug discontinuation criteria: (1) mRECIST CR determined by CECT/Gd-EOB-DTPA-enhanced MRI; (2) avascular by CEUS or pathologically curative resection; and (3) sustained within the normal range of AFP/AFP-L3 fraction/DCP levels for 12–24 weeks.
